# Social media use of adolescents who died by suicide: lessons from a psychological autopsy study

**DOI:** 10.1186/s13034-023-00597-9

**Published:** 2023-04-07

**Authors:** Elias Balt, Saskia Mérelle, Jo Robinson, Arne Popma, Daan Creemers, Isa van den Brand, Diana van Bergen, Sanne Rasing, Wico Mulder, Renske Gilissen

**Affiliations:** 1 Research department, 113 Suicide Prevention, Amsterdam, the Netherlands; 2 Orygen, 35 Poplar Road, Parkville, Vic 3052 Australia; 3 Centre for Youth Mental Health, The University of Melbourne, Melbourne, VIC Australia; 4 Child and Adolescent Psychiatry & Psychosocial Care, Amsterdam University Medical Centre (AUMC), Amsterdam, the Netherlands; 5 Child and Adolescent Psychiatry, GGZ Oost Brabant, Boekel, the Netherlands; 6 Department of Pedagogical and Educational Sciences, Faculty of Behavioural Social Sciences, University of Groningen, Groningen, the Netherlands; 7 Youth healthcare, Dutch Centre for Youth Health (NCJ), Utrecht, The Netherlands

**Keywords:** Social media, Suicide, Mental health, Adolescents, Psychological autopsy

## Abstract

**Background:**

while there are many benefits for young people to use social media, adverse effects such as cyberbullying, online challenges, social comparison and imitation may provoke and aggravate suicidal thoughts and behaviors. The influence of social media on mental health and suicidal thoughts and behaviours has been amply studied, but there is little empirical evidence for its potential role in adolescent suicides. The current study aimed to inform digital suicide prevention strategies by examining the meaning of social media in the lives of young suicide victims and elucidating the harmful and supportive effects of social media use on their wellbeing and distress.

**Methods:**

data were analyzed from a psychological autopsy study of 35 adolescents who died by suicide in the Netherlands (43% of all adolescents who died by suicide in that year). These were 18 girls and 17 boys. All were under the age of twenty years, with an average of seventeen years. Interpretative Phenomenological Analysis was performed of 55 semi structured interviews with peers and parents of the decedents.

**Results:**

young people benefitted from peer support and recovery stories. However, various themes were discussed relating to the harmful effects of social media, including dependency, triggers and imitation, challenges, cybervictimization and psychological entrapment. The themes of dependency and triggers and imitation were more salient in young females. A group of girls cultivated an online identity around their suicidal thoughts and behaviours. Next-of-kin, particularly parents, faced various challenges to talk to the adolescents about social media use, including technological illiteracy, online anonymity, and the youths’ closedness.

**Conclusions:**

based on the findings, we recommend education to stimulate the digital literacy of parents, health workers and educators, supporting conscientious social media use in young people, and extending the prevention of cyberbullying. We encourage future research to examine how virtual social networks may sustain suicidal thoughts and behaviour, and to further investigate the effectiveness of digital interventions, like moderated peer support and the use of positive role models.

**Supplementary Information:**

The online version contains supplementary material available at 10.1186/s13034-023-00597-9.

## Background

Suicide of young people is a major public health concern. On average, one young person under 20 dies by suicide every week in the Netherlands. It is a leading cause of death among adolescents aged 15–29 worldwide [1]. Increasing attention is directed towards identifying psychosocial risk factors of adolescent suicide. Cha and colleagues [2] report that the strongest lines of evidence are found for childhood maltreatment and bullying. Several psychological autopsy studies have additionally provided insights into psychosocial risk factors, including adverse life events and childhood trauma, and discussed the interplay between these factors [3–6]. The lives of youth are characterised by their family life, school, and social relations. Over the last decades, social media had played an increasingly prominent role in the development and lives of young people.

Social media is ubiquitous and intricately interwoven with the lives of people. For the purpose of this study, we adopted the definition of social media use provided by Ahmed and colleagues [7], whereby social media includes web and mobile platforms that allow individuals to connect with others within a virtual network, where they can share, co-create, or exchange various forms of digital content, including information, messages, photos, or videos. There are an estimated three billion social media users worldwide [8]. Whereas in adults, there is a clear dichotomy of online and offline social interactions, this distinction is less evident in young people. They grow up with plentiful online resources to interact and communicate with others, making them digital natives [9].

Literature is equivocal about the relation between social media use and mental health of adolescents. Earlier research by Mérelle and colleagues [10] in 21.053 Dutch adolescents indicates that problematic social media behaviour, defined by means of the Compulsive Internet Use Scale [11], is associated with conduct problems, hyperactivity, and sedentary behaviour. International evidence reports associations between the frequency and volume of social media use and sleep disturbance [12] as well as loneliness and depression [13, 14], and highlights the risks of upward social comparison [15–17]. Interestingly, in a recent study, adolescents themselves described social media as a key contributing factor to depressive thoughts [18]. Mental health problems, including depression, are among the strongest risk factors for suicide in adolescents [2]. There is also growing evidence for the relation between social media use and suicidal behaviours. Research from the US suggests that an increase in suicidal behaviours among adolescents may be partly attributed to an increase in new media screen time [19, 20]. Two meta-analyses by John and colleagues [21] and Nesi and colleagues [22] concluded that being the victim of cyberbullying is associated with self-harm, suicide ideation and suicide attempt. Several experts lastly express their worries about social media involvedness with suicide clusters [23–25].

Social media may conversely be beneficial to the wellbeing of young individuals, for example by increasing a sense of community and belonging [26–28], facilitating self-expression [29], and through peer support and the sharing of coping and recovering strategies [30]. Moreover, experts have discussed how social media platforms can be used to support adolescents suffering from suicidal thoughts. In a systematic review, Marchant and colleagues [31] urge that we exploit the benefits of social media, for example by facilitating crisis support, combatting isolation and loneliness, and delivering therapy. Some progress has been made in this regard. For example, Robinson and colleagues [32, 33] provide a tool to support young people to safely communicate about suicidal thoughts online, harnessing the potential of online peer support while mitigating the risk of harmful content and imitation of suicidal behaviours.

While several studies add to our understanding of the relation between social media, self-harm and even suicidal behaviours, there has been a paucity of empirical evidence for the relationship between social media use and suicide death in adolescents [34]. In previous work, we have touched on the topic of social media use among other risk factors for suicide, indicating that social media use had both positive and negative effects on the wellbeing of the deceased youths [6]. However, we are convinced that in depth analyses are warranted for a better understanding. The current study aimed firstly to elucidate the meaning of social media in the lives of adolescents who died by suicide, and to examine its effect on their wellbeing and distress. Secondly, we aimed to identify the needs of adolescents and next-of-kin relating to digital suicide prevention strategies by addressing how next-of-kin engaged young people about safe social media use, and the challenges they faced to talk to them about it.

## Methods

### Study design

This study used data from an observational, psychological autopsy study concerning adolescent suicide in the Netherlands [6]. The perceptions of individuals bereaved by the suicide of a young person provide a unique empirical perspective that is currently absent. As this field of enquiry is relatively unexplored, a qualitative approach can help support new theories and provide directions for future research [35]. One or both parents of the adolescent were the primary informants. Secondary proxy informants were family members and peers (next-of-kin), teachers and health care professionals. In the current research we did not include interviews with teachers and healthcare professionals, as their day-to-day contact with the adolescents was limited. In total, 81 adolescents aged 10–20 years old who lived in the Netherlands died by suicide in 2017. The sample included full word transcripts of 37 interviews with parents and 18 interviews with peers of 35 deceased adolescents. These were 18 girls and 17 boys. The age of the victims ranged from 14 to 19 years, with a mean age of 17 years. Participants were recruited through their general practitioner. Secondary informants were recruited through the primary informants. The sampling strategy is further detailed in the previous work [6].

### Materials

Interviews were semi-structured and had an average duration of two-and-a-half hours. The interview instrument was largely adapted from instruments of international psychological autopsy studies in Belgium, Norway, and the United Kingdom [3–5, 36, 37] and consisted of two parts. The first part involved a narrative description of the youth’s life, focusing on the respondents’ narrative and perceptions about key factors contributing to the suicide. The second part entailed five pre-identified topics: [1] adolescence, [2] physical and mental health and healthcare use, [3] social media use, series, games and imitation, [4] sexual orientation and gender identity, and [5] religion and ethnicity. These topics were informed by the research group and an advisory committee and supported by an extensive body of evidence concerning risk factors for suicide. While social media use was specifically addressed in one section of the interview, we expected the topic to be interwoven with other topics throughout the interview. Therefore, full word transcripts of the entire psychological autopsy interview were coded for the purpose of the current study. The interview has been attached with this article (additional file 1).

### Analysis

We performed post hoc explorative analyses on the psychological autopsy data. Interpretative Phenomenological Analysis was employed [38]. IPA was developed to investigate how individuals make sense of particular experiences and events. In this study, we analyzed the observations and experiences of next-of-kin, specifically the way they made sense of the meaning of social media to the lives and the suicides of the adolescents. We also address their perceptions about the impact of social media on the wellbeing and distress of the adolescents, and the challenges they faced to monitor and engage the adolescent about social media use. The choice of analysis was informed by two central research questions.

*What are the perceptions of bereaved individuals about supportive and harmful influences of social media use on the wellbeing and distress of adolescents who died by suicide?*

*How did next-of-kin talk to adolescents about safe social media use and what challenges did they face in engaging them?*

In the previous work, emphasis was placed on clustering and imitation, as opposed to the broader meaning of social media in the lives of the young people who died by suicide and its relation to the suicide. Post hoc analyses therefore progressed from the original coding. Deductive coding was used to determine the type of use and the different social media platforms frequented by the adolescents. Type of use was divided broadly into communication, lurking (looking at content without posting), and sharing content. We categorized social media platforms in line with the work of Chan-Olmsted and colleagues [39]. These are Social Networking sites (Facebook, Myspace, LinkedIn), blogging, and publishing media (Pinterest, WordPress), microblogging (Twitter), forums or message boards (Reddit, 4Chan, 9Gag), and media-sharing communities (YouTube, Instagram, Snapchat, Flickr). While we acknowledge that most media allow a form communication, we included communication media as a separate platform (WhatsApp, Facebook Messenger) because of the popularity of these media and their specific features.

Subsequent coding was inductive. Codes were formulated to reflect key themes relating to the meaning of social media to the young people, such as *a way to meet likeminded people* or *a place to share feelings*, the perceived effect of social media use, like *feeling understood by peers* or *becoming isolated* and, lastly, the dynamics between next-of-kin and the adolescent concerning social media use, such as *open conversation* or *restricting use*. The involved researchers [EB, IB, SM] reflected weekly on the coding approach, whereby the code list was discussed and edited. After coding approximately fifteen interviews, no major changes were made to the code list, and a definitive version of the list received consensus from the research team. The final code list is added as a supplement to this research (additional file 2).

## Results

### Social media use

All [35] deceased adolescents in our sample used social media. For fifteen adolescents, of whom twelve boys, social media was used primarily as a practical tool; to stay up to date about events or birthdays, or for school. They communicated through messenger apps but spent little time on social networking sites. For many young people [20], however, social media played an important role in their day-to-day lives. It was a way to stay in touch with peers, find people with similar interests, read about experiences of other young people, watch funny content, a safe space to (anonymously) vent their feelings, or to establish popularity by getting many friends or followers. All adolescents used communication media like WhatsApp and Facebook Messenger. Social networks [31 adolescents], and media sharing platforms [22 adolescents] were popular as well. Nine young persons frequented discussion forums or message boards. Eight out of these nine were girls visiting forums specifically related to mental health or eating disorders. Only a few [3 adolescents] used blogging and microblogging media.

### Harmful effects of social media use

Themes relating to the harmful effects of social media were dependency and excessive social media use, social comparison and distorted perceptions about mental health, triggers and imitation, cultivation of a suicidal identity, challenges and impulsive behaviours, and victimization and entrapment.

### Dependency and excessive social media use

Fourteen young individuals, who were often described by respondents as socially insecure, developed a dependency towards social media because they were anxious to be offline and miss out on conversations with peers or online content. Moreover, interactions with peers on social media affected their mood as much as real-life interactions, and they could become upset and distressed when they did not receive positive reinforcement from peers online.

*Parent: “The pressure of social media was significant. WhatsApp, but also Facebook and other platforms. The pressure of ‘befriending and unfriending’ and talking about each other through social media has had a very negative impact on her.”*

Insecurity and dependency were linked to excessive social media use. Eleven out of the fourteen adolescents actively responded to notifications throughout the entire day, even during dinnertime and late at night. Some spent up to eight hours a day browsing social media. While respondents did not attribute a direct effect of excessive social media use on suicidal thoughts or behaviours, they noted that it contributed to sedentary behaviours, feelings of anxiousness and loneliness, a decrease in face-to-face interactions, sleeping problems, and school problems.

*Parent: “He didn’t sleep well, was ruminating too much… he was locked in his thoughts. […] He felt like he did so much for this person [online friend], and he get so little in return. That really bothered him, and he could no longer focus when he was in school.”*

### Social comparison and perceptions about mental health

The content posted by peers and role models presented norms for mental health and happiness that distorted some of the decedents’ perceptions about their own mental health. The emphasis on achievements, events and positive emotions presented a stark contrast with suffering from suicidal thoughts and created pressure. By consistently comparing their lives with the seemingly perfect lives of peers and influencers online, feelings of being different were reinforced.

*Parent: “We tried to normalize it. ‘Honey, whatever you’re feeling, and those thoughts you have, a lot of people your age have those. It may seem from the outside like everyone has it made, everyone has a great live, everyone looks nice, everyone has nice clothes, money. But that is not reality, that’s just a shell. Like you see on social media, you know, that’s not life.”*

### Triggers and imitation of suicidal behaviours

For a group of thirteen adolescents, of whom twelve girls, there was a thin line between supportive and triggering online networks. By trigger we refer to online content that elicited feelings of distress in the adolescent. These adolescents actively looked for recognition and support in online communities but became entangled in communities where triggering content was openly shared. Content ranged from depressing quotes and *memes* to graphic depictions of self-harm and suicide. Parents berated how easy it was to find information about how to die by suicide on discussion forums and social networking platforms. Some respondents feared that adolescents may have become preoccupied with self-harm and suicidal behaviours by being consistently confronted with other peoples’ experiences in the matter.

*Parent: “There is a world of websites filled with negative content. Experiences of people. […] And then there are these algorithms. If you frequently search for negative things on Instagram, then Instagram will show you more negative content. That not just makes it harder; it makes it look like that is all there is. Because that is the only thing that comes up.”*

*Parent: “On Instagram, you can follow a certain topic. She was taken in with depression as a topic. All these posts and memes about depression, she read those a lot. It occupied her. She also searched for ‘suicide.’ Continuously feeding those negative thoughts through social media. […] It is a downward slope. If you only surround yourself with negativity, that is all you can feel.”*

This theme was most salient in eight girls who had been admitted into a healthcare facility. The girls were exposed to suicidal behaviours of fellow residents in real life. Parents described that WhatsApp groups were formed to support fellow residents, but that these channels were often used by young persons to *vent;* to share their thoughts and feelings. There was insufficient engagement and moderation of social media use in inpatient settings. While we found no conclusive evidence of clustering of youth suicides in our sample, next-of-kin warned that suicide-related content on social networking and media sharing platforms may promote imitation of suicidal behaviours.

*Parent: “She also had this recovery account on Instagram. A lot of girls with an eating disorder or what not have these accounts. She allowed me to watch it from time to time, but I wasn’t allowed to follow her. She regularly showed me things. […] That they show each other wounds from self-harming, but also posts like ‘I don’t feel like living anymore, I quit.’ The things they had done. I saw all those things.”*

*Peer: “Sometimes it becomes a competition. Everything is competitive. How thin can you become? How deep can you cut? Those things. Who is the most disturbed? I think [name] did not feel a need to be the most disturbed one. But I believe she was the most disturbed one without even trying.”*

### Cultivation of a suicidal identity

Another theme emerged for a small group of adolescents who communicated often and openly about their suicidal thoughts. They developed what a peer referred to as a suicidal identity. This transcended frequent suicide-related communication, as it maintained that the young person became so entangled with their own suicidal thoughts and behaviours, that they found it hard to imagine their life without them. The adolescents for whom this theme applied were afraid of who they would be once they recovered because their mental health problems provided them with a sense of belonging and identity. The peer of a young girl explained.

*Peer: It can be quire harmful, when you’ve built a life like that, because who will you be when that’s gone? […] Who will I be once I recover? If I just complete an education, who am I, what will it be that makes me interesting, what will it be that defines me? […] But I think that social media, like Tumblr and Instagram and all those platforms that [name] was on, that those helped her hang on to that identity, anchored it. ‘This is what I am, and this is what makes me, me.’ While there was so much more that made her, her.*

Young people cultivated this identity through online peer-to-peer interactions and reinforcement, seeking either sympathy or disapproval. Next-of-kin worried that cultivating an identity around suicidal thoughts and behaviours, and specifically the fear of letting go of this identity, aggravated suicidal thoughts and hampered help seeking and openness to treatment and recovery.

### Challenges

Challenges are online phenomena where (often young) people challenge one another to carry out specific tasks, sometimes sharing videos of themselves performing these tasks through social media. Five adolescents in our sample had participated in challenges. Three of these challenges involved physical harm. One girl suffered from intoxication following excessive water intake, following a challenge to drink as much water as possible to lose weight. One boy participated in a choking challenge, where a person asphyxiates themselves until nearly passing out for the thrill.

A young girl was known to have shown interest in a suicide-related challenge: the Blue Whale challenge. This challenge entails that a person follows a set of activities, imposed upon them by a curator. The activities are harmless at first, like setting an alarm in the middle of the night, but gradually evolve to tasks such as instigated self-harm. The final task is to end one’s own life. A girl talked about taking part in this challenge with a friend and had reached out to a curator online. As far as the respondents know, however, she never completed any of the more dangerous tasks in the challenge.

*Peer: “She participated in a challenge, the Blue Whale challenge. You need to do different tasks. […] I know that it was directed at depressed people. I don’t have any examples, but these tasks were nothing like an ice bucket challenge or something.”*

None of the respondents believed that the suicide could be attributed to participating in challenges, but they did acknowledge how challenges may incentivize impulsive behaviours in at-risk adolescents, resulting in a disregard for the consequences of potentially harmful behaviours.

*Parent: She was regularly active on social media. At some point, she had an app, it was called Kik. You have all these young people posting stuff that gives you a thrill. […] She was so impulsive and impressionable. Just to be part of it. She was so impressionable by all that happened around her.”*

### Victimization and entrapment

Cyberbullying was reported in four cases. These adolescents received harmful messages or were threatened with violence through online media. Bullying could include the encouragement of suicidal behaviours. Two young people were pressed by peers to engage in self-harm or a suicide attempt. A young girl made a reference to the bully briefly before her suicide, while the event took place many years before, suggesting a lasting impact.

*Parent: “[A boy in class] asked her: when will you commit suicide? And we found out, later, that this did not happen once, it was asked to her twenty times a day. […] A few days before [she took her life], she told her mother: ‘I can still see that boy sometimes’.”*

Five girls and one boy were victim of cybervictimization, specifically online sexual transgressive behaviour. Online relations with the perpetrators started mutually but quickly developed into an unequal relation. The adolescents received unsolicited proposals, pictures, or videos, or were themselves forced to send pictures or videos involving nudity or sexual acts. In most cases, these were single events, and not associated with the suicide of the adolescents. However, two adolescents had traumatic experiences following cybervictimization which the respondents associated with the suicide. One boy had been the victim of online catfishing and was sexually assaulted by an older man. A young girl was the victim of sextortion by fellow residents of a care facility. They threatened to release nude pictures and videos of her on the web. Feelings of entrapment were often reported for bullied and victimized adolescents.

*Parent: “I know they were allowed to be on the computer for half an hour a day, and they could go on Facebook, but a lot more happened there, because in that time she sent nude pictures of herself, and videos too, under pressure. Some inpatient boys told her: ‘send me some nudes of yourself, or I will tell the staff that you have cigarettes and a lighter in your room.‘ And that escalated.”*

### Supportive use of social media

Next-of-kin of ten adolescents emphasized the supportive aspects of social media. Supportive elements of social media as perceived by respondents were recognition and understanding, peer support, recovery stories and coping strategies, and healthy networks.

For adolescents, social media presented a tool to seek recognition and understanding, which they found more easily in communities of peers with similar problems. Notably, the feeling of not being understood in the offline world strengthened their urge to seek interactions with peers online. They sought help and support from role models and peers to deal with their psychological problems, like coping strategies that helped others and recovery stories. It helped normalize their suicidal thoughts and made them feel less alone in their struggles. Some adolescents felt empowered when using social media to share their experiences to help others.

*Peer: “That is also what she tried to express. You shouldn’t only look at the negative things, because that will not help you recover.”*

A specific theme relating to supportive effects was found in a group of girls in inpatient settings. Social media were a necessity for them to sustain relations with friends and family outside of the care facility. These adolescents witnessed the daily distress of peers, and many had witnessed suicidal behaviours of fellow residents. Online interactions with friends and family outside the clinic firstly made them feel looked after and, secondly, counterbalanced the exposure to psychiatric problems and systematic suicidal behaviours of fellow residents.

### Experiences with monitoring and engaging adolescents about social media

Interestingly, although five peer respondents expressed worries about the impact of social media to vulnerable adolescents in the interviews, none of them reported to have engaged their fellow peers about such concerns. By contrast, at least nineteen parents had often tried to engage the adolescents about their social media activities because they worried about the harmful effects and the impact on the youth’s mental health. Adolescents were naturally reserved about their social media use towards their parents, which caused parents to be ill informed about online behaviours. Several parents mentioned feeling frustrated that while they started the conversation with an unjudgmental attitude, adolescents refrained from open conversation. They furthermore experienced various challenges that made them feel inapt to address the issue properly. Common themes included unfamiliarity, secrecy and anonymity, and restriction and resistance.

### Unfamiliarity

Most parents were not familiar with the functionalities of social media platforms and unaware about developments and trends on social media. Consequently, engaging an adolescent was difficult because parents did not know what kind of questions would incite open conversation. Illiteracy towards the gaming community and associated media platforms was specifically addressed, including game chat servers and forums.

*Parent: That was an unknown world to us. I learned about it. ‘Are you behind your computer* again*? See some friends!’, I would say. But that’s just what they do with each other. He had a lot of friends online, including neighbourhood friends. […] They organize tourneys. They would game the whole weekend. His tiredness was a problem because they would go on all night”*.

*Parent: There are other chat programs for gaming about which we know little. He was on the dark web, those kinds of places. He had a vast number of chat communities with gamers from all over the world.”*

### Online anonymity

Respondents of twelve cases reflected on the secrecy and anonymity of social media behaviours. Young persons were adept at hiding their online activities, ranging from simply deleting their browser or search engine history to using virtual private networks. Several adolescents created a second account on social media platforms, under another name. At least five young girls used this account to enter communities anonymously and to share explicit suicide-related communications. Respondents note that these young girls wanted to prevent that suicide-related content would reach next-of-kin in real life because they were afraid of judgment or consequences. A few parents were so anxious to be knowledgeable, that they created a secret account themselves to monitor the youth or asked a sibling to provide them with information about suspicious online activities of the youth.

### Restriction and resistance

To maintain control and involvement, parents monitored online activities, or set time limits for internet use. However, active restricting often did more harm than good, explained parents. Restriction triggered a strong, emotional response, and adolescents took extensive measures to stay connected. To illustrate, one girl bought a new phone every time her parents took her phone.

*Parent: “At some point we took her phone away, turned out she had a second one. I think she even got a third one. There was no stop to it. The [restriction] had mostly to do with her running away [from the clinic]. And to make sure she finally got a good night’s rest instead of all those notifications.”*

## Discussion

The current study revealed how bereaved individuals made sense of the meaning of social media in the lives of adolescents who died by suicide, and how they perceived its impact on the youth’s wellbeing and distress. Notably, a direct relation between social media and the adolescents’ suicide was only perceived for victims of severe cyberbullying or victimization. The major impact of bullying on youth wellbeing has been stressed before [21]. Particularly concerning is a form of bullying that involves the encouragement of suicidal behaviours. Adolescents felt they could not escape their bullies and were simultaneously pressed towards a deadly end of their victimization. A parallel may be observed with psychological entrapment, which has been proposed by O’Connor and Kirtley [40] as an important psychological risk factor for suicide. Experts of law have debated whether cyberbullying should be criminalized but acknowledged that proper education has more merit [41, 42].

Most of the other identified themes align findings from earlier research about the relation between social media use and suicidal behaviours, including triggers and imitation [5, 23–25] and upward social comparison and distorted perceptions about mental health [15, 16]. While next-of-kin associated participation in challenges with impulsive traits and risk behaviours, no relation with the suicide was assumed in any case. Social media was predominantly perceived not to cause but to amplify negative thoughts and behaviours in adolescents, particularly in those who were described as insecure, vulnerable, and lonely. Huang and colleagues [43] propose in their *displacement* hypothesis that social media use can be deteriorative when the user perceives little social support in their own environment and follows strangers online to seek understanding and recognition there instead. Indeed, some of the identified harmful effects of social media use for adolescents in our sample could be traced back to the motivation to use social media and the manner of use. Girls in our sample also appeared to be at higher risk for the harmful dynamics of social media use. They frequented social networking sites because they felt misunderstood or unheard by family and friends. Many boys instead used social media as a practical extension of their offline life. Moreover, the themes *dependency*, *triggers and imitation*, and *suicidal identity* were most salient in the female decedents. While they reportedly did find recognition online, they were consequently exposed to suicide-related content, such as graphic images of self-harm, and detailed information about how to end their life. These findings corroborate earlier research, indicating that harmful dynamics on social media affect the mental health of young females more strongly [44, 45].

Bereaved individuals worried that technological features of social media that produce depression or suicide-related content based on former online behaviours through algorithms may facilitate the development and cultivation of a suicidal identity. Constructing an identity that is informed by mental health problems has been described in other contexts, including substance addiction [46] and eating disorder [47], and a cross-sectional study indicates that a minority of adolescents reported to self-harm to reinforce their group identity [48]. Online reinforcement of an identity around suicidality may sustain suicidal thoughts and behaviours and have profound implications for help-seeking and treatment, but this requires further enquiry. Moreover, consistent confrontation with graphic content of self-harm and suicide on social media might increase what is called acquired capability for suicide through desensitization. Acquired capability refers to the degree to which an individual is able to enact a lethal suicide attempt [49]. Developing acquired capability results from repeated exposure to painful and provocative life events which habituate an individual. Most evidence points out that an acquired capability is increased by physical experiences with pain, particularly suicide attempts [50–52]. However, Smith and Cukrowicz [53] suggest that visual stimuli may also affect acquired capability through neuropsychological processes habituating fear.

This study offers first empirical findings about the experiences and challenges faced by next-of-kin to monitor and address social media use of adolescents who died by suicide. Peers were knowledgeable and had a nuanced perspective towards social media use compared to parents but typically did not engage their peers about the risks of social media use, even though they did discuss harmful effects in the interviews. Parents tried to engage the adolescents about their concerns, but the interviews revealed the various challenges faced by them. Restriction was reported to be counterproductive for adolescents in our sample. Recent evidence is equivocal about social media restriction. Fardouly and colleagues [54] report that children whose parents reported greater control over social media behaviours reported better mental health. However, Hampton and colleagues [55] warn that “*restrictive media parenting practices or digital inequalities may rob adolescents of experiences that would otherwise be protective of self-esteem.”* Either way, the implications and recommendations of earlier research have often suggested that we equip parents with the knowledge and skills to engage their children about harmful social media use (see for example Procentese et al. [56]). While this is both understandable and important, we must also acknowledge that adolescents may never be completely open towards parents about their online activities and its impact on their wellbeing because they value their autonomy. Furthermore, adolescents are adept at hiding their online activities as digital natives and have mobile phones which allow their online activities to take place outside home settings. We therefore recommend exploring additional strategies that appeal to the youth’s own advocacy and self-efficacy instead, like the *#chatsafe* initiative, which was co-designed together with adolescents [32, 33]. Additionally, we may generate awareness about harmful social media use through peer networks and equip adolescents with handles for conscientious, responsible social media use.

### Limitations

Some limitations of this study must be noted. Firstly, no control sample has been included. Therefore, we cannot conclude if the themes addressed in this research are unique to adolescents who died by suicide, or that other adolescents experience similar problems. Secondly, in the psychological autopsy [6] we focused on factors contributing to the suicide and prioritized risk factors as opposed to protective factors. Next-of-kin respondents may have therefore overestimated the negative impact of social media out of a desire to find reasons why their loved one died by suicide, prompted by the questions in the instrument. This could have obscured supportive effects of social media use and its potential for prevention [30, 31]. Thirdly, earlier research has stressed the risk of recall bias in psychological autopsy studies. As we interviewed proxy informants up to 2.5 years after the suicide, it is reasonable to assume they have forgotten some details relating to the suicide [57]. However, during the interview, several respondents showed and scrutinized the social media activities of the decedent on their phone, to illustrate the nature of their perceptions. Moreover, we found that respondents provided detailed accounts of day-to-day activities, including what they knew about social media use. This may be explained by research of Assink [58] who suggest that negative life events can be more consistently recollected compared to neutral or positive events. Triangulation can further reduce the risk of recall bias. Peers of eighteen deceased adolescents were interviewed. Looijmans and colleagues [59] have shown that including peers in psychological autopsies is recommendable when examining those facets of deceased young people’s lives about which parents have less in-depth knowledge, including relationships, school problems, and social media use. Lastly, this study has not specifically addressed the role of online influencers, who may effectively reach youths with positive reinforcement, sensitize young people about mental health problems in the online environment, and challenge the negative effects of upward social comparison [60].

## Conclusions

This has been one of the first studies to provide empirical evidence for the meaning of social media use to the life and the suicide of adolescents and its impact on their wellbeing and distress. The study thereby delineates some of the needs of young people suffering from suicidal thoughts and next-of-kin in relation to social media use and offers handles for digital prevention strategies. The supportive effects of social media can become overshadowed when social media are used as a replacement, rather than a supplement, of family and friends’ support and professional care. To foster safe social media use, we may equip young people and next-of-kin with knowledge and skills but must also bear in mind the responsibility of social media platforms and policy makers to create a safer online environment. Specific attention should be paid to young females who are sensitive to harmful dynamics on social media. Cyberbullying and victimization require rigorous prevention efforts. In doing so, we can harness the potential of social media for suicide prevention efforts and mitigate the risks of social media use for adolescents with suicidal thoughts.

## Electronic supplementary material

Below is the link to the electronic supplementary material.

Supplementary Material 1

Supplementary Material 2

## Data Availability

Interview data cannot be shared publicly because of ethical restrictions: the dataset contains potentially identifying and sensitive information and the Medical Research Ethics Committee (MREC) of Amsterdam UMC has imposed this restriction (registration number: 2018.651 – NL68348.029.18). Contact information: ln.cmuv@ctem; https://www.vumc.nl/research/overzicht/medisch-ethische-toetsingscommissie.html. Data may be made available upon reasonable request. Contact the author (EB) for enquiries.
