# Supplementary material for: Social media use of adolescents who died by suicide: lessons from a psychological autopsy study
Source: Child Adolesc Psychiatry Ment Health. 2023 Apr 7;17:48. doi: 10.1186/s13034-023-00597-9 (PMC10082488; doi:10.1186/s13034-023-00597-9)
Supplement: Supplementary file 2 — Supplementary Material 2 [file 13034_2023_597_MOESM2_ESM.docx]

Social media use of adolescents who died by suicide

**Code list**

Authors: Elias Balt, Isa van den Brand, Saskia Mérelle

**SM_usetype**

Sm_usetype_communication

The youth used social media to communicate with other people

Sm_usetype_lurking

The youth used social media to look at content of other people

Sm_usetype_posting

The youth used social media to share posts in the form of videos, images or text

**SM_platform**

Platform_social_networking

The youth frequented Social Networking sites (Facebook, Myspace, LinkedIn)

Platform_publishing_media

The youth frequented publishing media (Pinterest, WordPress)

Platform_microblogging

The youth frequented microblogging media (Twitter)

Platform_forums_message_boards

The youth frequented forums or message boards (Reddit, 4Chan, 9gag)

Platform_media-sharing_communities

The youth frequented media-sharing communities (YouTube, Instagram, Snapchat)

Platform_communication_media

The youth used sec communication media (WhatsApp, Facebook Messenger)

**SM_meaning**

All codes relating to the meaning social media had in the lives of the young people who died by suicide, as reported by respondents.

SM_meaning_practical

Social media was a practical tool to the young person. (s)he used it to organize school work, stay up-to-date about events

SM_meaning_peer_interaction

Social media was a way to interact with peers from real life, including messaging and sharing content with each other.

SM_meaning_share_experiences

Social media was a way to share experiences with people online, both peers and strangers. This included looking for feedback. The youth expressed his/herself online through posting.

SM_meaning_meeting_people

Social media was seen as a way of meeting new people, outside of the regular social circles of school and neighborhood friends and family.

SM_meaning_entertainment

Social media was a place to look for easy entertainment.

SM_meaning_status

Social media was a way to establish a certain status or level of popularity. To find a place in a perceived ranking order. In some cases closely related to the negative impact of social comparison.

SM_shared_interests

Social media was used by the youth to explore interests, and find things the young person liked to see or do.

SM_safe_anonymous

Social media represented a safe space for the young person, where they could be whoever they wanted to be, whether anonymous or not.

**SM_harmful_impact**

All codes describing themes concerning the harmful impact of social media use on the wellbeing and distress of the young people.

SM_wellbeing-_excessive

Respondents report excessive social media use. Excessive in this instance is not defined by objective parameters, but rather the subjective interpretation of the respondent as based on their observations.

SM_wellbeing-_dependency

Youths developed a dependency on social media according to respondents. This indicates they found it difficult to go without social media, and could become easily distressed when they were not able to use social media.

SM_wellbeing-_triggers

Youths were confronted with triggering content on social media, including graphic violent or sexual content, or harmful mental health-, or suicide-related content.

SM_wellbeing-_preoccupation/desensitization

Young people became preoccupied with suicide-related content, and increasingly searched for it. Motivation for this preoccupation could differ, ranging from being in a social network with many peers with mental health problems to algorithms reinforcing search behaviours and shown content.

SM_wellbeing-_comparison

Youths were affected by a comparison of their own social life and emotional state with other people online, both peers and influencers. It affected their perceptions about their own mental health.

SM_wellbeing-_suicidal_identity

Social media was used to develop, cultivate and/or anchor an unhealthy identity revolving suicidal thoughts or behaviours by seeking validation and feedback from people online.

SM_wellbeing-_imitation

Youths copied harmful behaviours from online examples

SM_wellbeing-_imitation_challenges

Youths copied harmful behaviours from online examples, specifically from online challenges.

SM_wellbeing-_victimization and entrapment

Young people were victimized by peers or strangers online, in the form of bullying or sexual transgressive behaviours. Strong feelings of entrapment were noted for online victimization, because of the omnipresence of social media.

**SM_positive_impact**

All codes describing themes concerning the supportive impact of social media use on the wellbeing and distress of the young people.

SM_wellbeing+_ venting

Social media was a valuable tool to vent feelings. This code was always coded together with SM_meaning_sharing_experiences. By venting their feelings online, they combatted rumination. However, it must be noted that venting could help, but could also induce negative responses and lead to cybervictimization or being exposed to a lot of triggering content.

SM_wellbeing+_understanding

Youths found understanding online from peers or strangers, which helped normalize feelings of sadness or anxiety, and reduce their distress. Notably, youths for whom this code was applied found it hard to find understanding in the offline world.

SM_wellbeing+_stay_in_touch

Youths used social media to stay in touch with persons whom they found hard to reach in real life, due to circumstances (e.g. physical distance, inpatient admission).

SM_wellbeing+_recovery

Reading about examples of recovery and coping of likeminded peers provided valuable handles to the youth to deal with their own mental health problems.

**SM_dynamics**

All codes reflecting on the interaction between next-of-kin and the adolescent about their social media use, including open conversation, monitoring and restrictions, and the challenges they faced in these dynamics.

SM_dynamics_unfamiliarity

Next-of-kin were unfamiliar with social media in terms of functionalities, type of use, or trends. This prevented them from engaging in open conversation

SM_dynamics_openness

Youths were open to talk about social media use with next-of-kin, which stimulated involvement and increased next-of-kins’ understanding of the meaning of social media for the adolescent.

SM_dynamics_anonymity

Youths used the anonymity of online resources to avoid being monitored by next-of-kin. This included creating secret accounts or using another phone.

SM_dynamics_monitoring

Segments of text describing how the next-of-kin showed an interest or monitored the social media behaviours of the young person, including challenges faced to monitor these behaviours.

SM_dynamics_restriction

Any experiences shared by next-of-kin respondents relating to restricting access to the internet or social media specifically.
